# Therapy-induced methionine competition in bladder cancer: MAT2A-SAM axis remodeling as a metabolic immune checkpoint

**DOI:** 10.3389/fimmu.2026.1965813

**Published:** 2026-10-22

**Authors:** Chengyuan Zhang, Zhe Zhang, Jianbin Bi

**Affiliations:** Department of Urology, The First Hospital of China Medical University, Shenyang, China

**Keywords:** bladder cancer, CD8+ T cells, cisplatin resistance, immunotherapy, MAT2A, methionine metabolism, SLC7A6, YTHDF1

## Abstract

Bladder cancer is increasingly treated with platinum chemotherapy, immune checkpoint blockade, and antibody-drug conjugates, but treatment resistance remains common. Methionine connects nutrient availability with S-adenosylmethionine (SAM) production, methylation, redox homeostasis, and lymphocyte function. In cisplatin-resistant tumor cells, MAT2A stabilization sustains a SAM-dependent program of stem-like renewal and drug resistance. Concurrently, increased SLC7A6-associated methionine uptake can create a tumor methionine sink that lowers methionine and SAM in CD8+ T cells, weakens H3K79me2-STAT5 signaling, and impairs effector function. Separately, methionine-sensitive YTHDF1 signaling suppresses RIG-I-dependent innate sensing and increases PD-L1 translation. A layered metabolic immune checkpoint is proposed to explain how these convergent but non-equivalent mechanisms link treatment-selected tumor fitness, nutrient competition, and reduced immune visibility. The framework exposes a context-dependent therapeutic paradox: methionine restriction may weaken tumor adaptation but compromise T-cell fitness, whereas SAM restoration may support antitumor immunity while also fueling malignant cells. Therefore, treatment-state biomarkers should guide compartment-selective targeting and intervention timing.

## 1 Introduction

Bladder cancer is a heterogeneous urothelial malignancy in which treatment is shaped by disease stage, renal function, and tolerance to systemic therapy (1, 2). Platinum chemotherapy remains central to perioperative management and selected metastatic treatment, while pembrolizumab, avelumab maintenance, and enfortumab vedotin plus pembrolizumab have broadened systemic options (3–5). Even so, durable disease control is not universal. Primary and acquired resistance, stromal exclusion, and weak CD8+ T-cell effector programs continue to limit therapeutic benefit (6, 7).

The persistence of resistance has pushed the field beyond classical receptor-ligand checkpoints. Chronic antigen exposure can drive T-cell exhaustion, but antigen is not the only pressure operating within a tumor. Mitochondrial insufficiency, hypoxia, and competition for nutrients can reduce the functional fitness of infiltrating lymphocytes even when PD-1/PD-L1 signaling is blocked (8–10). Tumor metabolism is therefore not simply a consequence of rapid proliferation. It is an active program through which cancer cells preserve survival and reshape the immune microenvironment. Methionine metabolism sits at the intersection of nutrient availability, protein synthesis, methyl-donor balance, and epigenetic regulation.

In contrast to this broad pathway-centered perspective, this Mini Review uses a bladder cancer-specific framework organized by treatment state, cellular compartment, and functional consequence. It distinguishes cisplatin-selected MAT2A dependence and SLC7A6-associated methionine competition (11) from a parallel methionine-sensitive YTHDF1 program that reduces immune visibility (12). YTHDF2 defines an additional RIG-I-suppressive branch that has not yet been linked directly to methionine availability (13). We use “metabolic immune checkpoint” to describe a proposed layered metabolic state. In this state, tumor-cell metabolic adaptation, methionine competition between tumor and immune cells, and immune-cell dysfunction may converge to limit antitumor immunity. The term does not denote a single receptor–ligand interaction, such as PD-1/PD-L1, or imply that these mechanisms must form a single linear pathway. Throughout, we distinguish direct bladder-cancer evidence from cross-tumor mechanistic support and translational inference.

## 2 The methionine-SAM axis: a shared metabolic currency

One-carbon metabolism integrates the folate and methionine cycles with nucleotide synthesis, redox defense, and methylation (14–16). MAT2A catalyzes the conversion of methionine and ATP to SAM, the principal methyl donor for DNA, RNA, histone, protein, and lipid methylation. SAM-dependent reactions generate S-adenosylhomocysteine, which is converted to homocysteine and then remethylated to methionine or directed toward transsulfuration. The methionine cycle is therefore not an isolated pathway, but a metabolic control system connecting extracellular nutrient availability with intracellular methylation capacity (**Figure 1**).

**Figure 1:**
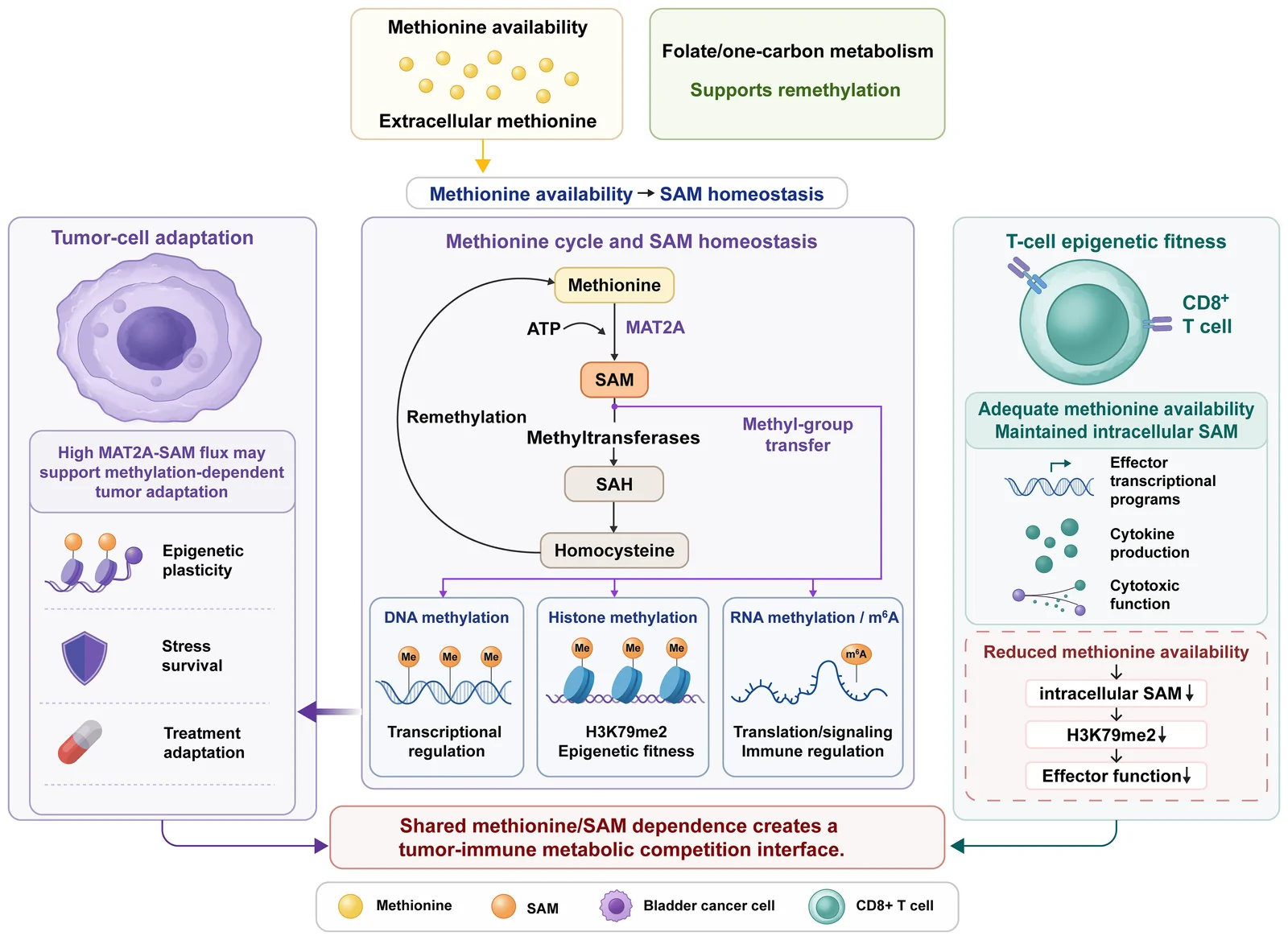
The methionine cycle and MAT2A-SAM-dependent epigenetic control. MAT2A converts methionine and ATP to SAM, which supplies methyl groups for DNA, histone, and RNA methylation. Methyl-group transfer generates SAH, which is hydrolyzed to homocysteine by S-adenosylhomocysteine hydrolase. Folate-dependent remethylation regenerates methionine through methionine synthase, with 5-methyltetrahydrofolate as the methyl donor and vitamin B12 as a cofactor. Increased MAT2A-SAM flux may support methylation-dependent tumor-cell adaptation. In CD8^+^ T cells, adequate methionine availability and intracellular SAM support epigenetic and effector functions. Reduced methionine availability can lower intracellular SAM and H3K79me2, impairing effector function. The schematic highlights shared metabolic requirements and selected methylation-dependent functions; their consequences depend on cellular context. Transsulfuration, methionine salvage, and other branches are omitted for clarity. ATP, adenosine triphosphate; MAT2A, methionine adenosyltransferase 2A; SAM, S-adenosylmethionine; SAH, S-adenosylhomocysteine; CD8, cluster of differentiation 8; H3K79me2, histone H3 lysine 79 dimethylation; m^6^A, N^6^-methyladenosine; Me, methyl group.

Cancer cells use this system to maintain relatively stable adaptive states. Methionine availability changes intracellular SAM, the SAM:S-adenosylhomocysteine ratio, histone methylation, transcription, and protein synthesis, while mTORC1 can induce MAT2A and couple SAM production to m^6^A-dependent translation (17, 18). MAT2A is therefore more than a biosynthetic enzyme; it is a metabolic-epigenetic node through which tumors translate nutrient status into stemness, stress tolerance, and treatment survival.

Activated T cells depend on the same metabolic currency. Antigen-receptor signaling induces amino-acid transporters, and SLC7A5 is required for efficient methionine uptake and methyl-donor generation during T-cell activation (19). Methionine availability also supports the epigenetic reprogramming of helper T-cell responses (20, 21). Because tumor cells and lymphocytes need the same substrate for different purposes but do not have equal access, this shared dependence creates a central imbalance in the tumor microenvironment. Nutrient partitioning is cell programmed rather than passive (22). By increasing methionine acquisition, tumor cells can preserve their epigenetic flexibility while lowering the metabolic threshold at which infiltrating T cells become dysfunctional.

## 3 Methionine remodeling links tumor adaptation, nutrient competition, and immune escape

### 3.1 Cisplatin selects a MAT2A-dependent survival state

Cisplatin-resistant bladder cancer cells acquire adaptive programs extending beyond DNA-damage repair. Epigenetic regulation (23–25), altered amino-acid and lipid metabolism (24, 26), antioxidant defense (27), and oxidative phosphorylation (28) contribute to the resistant state. Within this wider adaptation, integrated metabolomic and proteomic analysis identified MAT2A-regulated methionine metabolism as a major vulnerability of cisplatin-resistant bladder cancer cells (11).

Mechanistically, circARHGAP10 acts as a scaffold linking MAT2A to the E3 ligase TRIM25, thereby accelerating MAT2A ubiquitination and proteasomal degradation. Loss of circARHGAP10 in resistant cells reduces this interaction, stabilizes MAT2A, and preserves SAM-related methylation, stem-like renewal, and cisplatin survival. Restoring circARHGAP10 or suppressing MAT2A weakens this program and resensitizes resistant cells to cisplatin (11). Therapy therefore does not merely leave behind cells that happen to survive. It selects a metabolic state in which methyl-donor supply and epigenetic plasticity become part of the resistance machinery.

### 3.2 SLC7A6 turns tumor adaptation into methionine competition

The metabolic adaptation extends beyond the tumor cell. Conditioned medium from cisplatin-resistant bladder cancer cells impairs CD8+ T-cell function, yet tumor-cell MAT2A silencing alone does not restore it. Instead, resistant cells use SLC7A6-associated uptake to lower extracellular methionine and reduce intracellular methionine and SAM in neighboring CD8+ T cells (11), providing the bridge to the immune compartment.

The result is a functional tumor methionine sink in which reduced methionine lowers SAM, H3K79me2, and STAT5 signaling in CD8+ T cells. IL-2, IFN-γ, and granzyme B also fall as PD-1 increases, whereas methionine repletion partly restores these readouts and supports a causal link to the exhaustion-like phenotype. SLC7A6 knockdown restores these programs, while inhibition of L-type amino-acid transport with BCH enhances the effect of PD-L1 blockade in an immunocompetent MB49-CR mouse model (11). SLC7A6-associated methionine capture can therefore convert a tumor-cell metabolic advantage into impaired CD8+ T-cell signaling and function.

### 3.3 Methionine-rich tumor cells become less visible to immunity

RNA methylation and innate immune sensing define a distinct tumor-intrinsic branch in which high methionine availability increases m^6^A-linked YTHDF1 expression. YTHDF1 promotes the loss of RIG-I transcripts and attenuates the RIG-I/MAVS/TBK1/IRF3 pathway, reducing type I interferon signaling. In parallel, YTHDF1 interacts with eIF5B and enhances PD-L1 mRNA translation (12). The same methionine-rich state that supports tumor growth can therefore suppress antiviral-like innate sensing while strengthening a canonical checkpoint ligand.

In MB49 models, methionine restriction or YTHDF1 suppression combined with immune checkpoint inhibitors (ICIs) improves tumor control. Local methionine depletion also increases immune infiltration and prolongs mouse survival. Multiplex immunofluorescence in patients receiving immunotherapy provides associative support for the YTHDF1–RIG-I–PD-L1 module (12). This branch adds a second form of metabolic immune escape: SLC7A6 limits lymphocyte nutrient supply, whereas YTHDF1 reduces tumor visibility and strengthens resistance to immune attack.

### 3.4 From metabolic adaptation to a layered immune checkpoint

The available studies support convergence at the level of biological outcome but do not establish a single linear pathway. The circARHGAP10-TRIM25-MAT2A axis preserves the resistant tumor cell; SLC7A6-associated uptake restricts methionine available to CD8+ T cells; and the methionine-YTHDF1 program suppresses innate sensing while enhancing PD-L1 translation. We therefore propose a layered metabolic immune checkpoint connecting tumor survival, nutrient allocation, T-cell fitness, and immune visibility through methionine metabolism. A key unresolved question is whether these components co-occur, and in what temporal order, in human tumors. **Figure 2** summarizes the proposed convergence of the MAT2A-SAM and SLC7A6-associated methionine-competition programs; YTHDF1-dependent regulation of immune visibility is considered separately.

**Figure 2:**
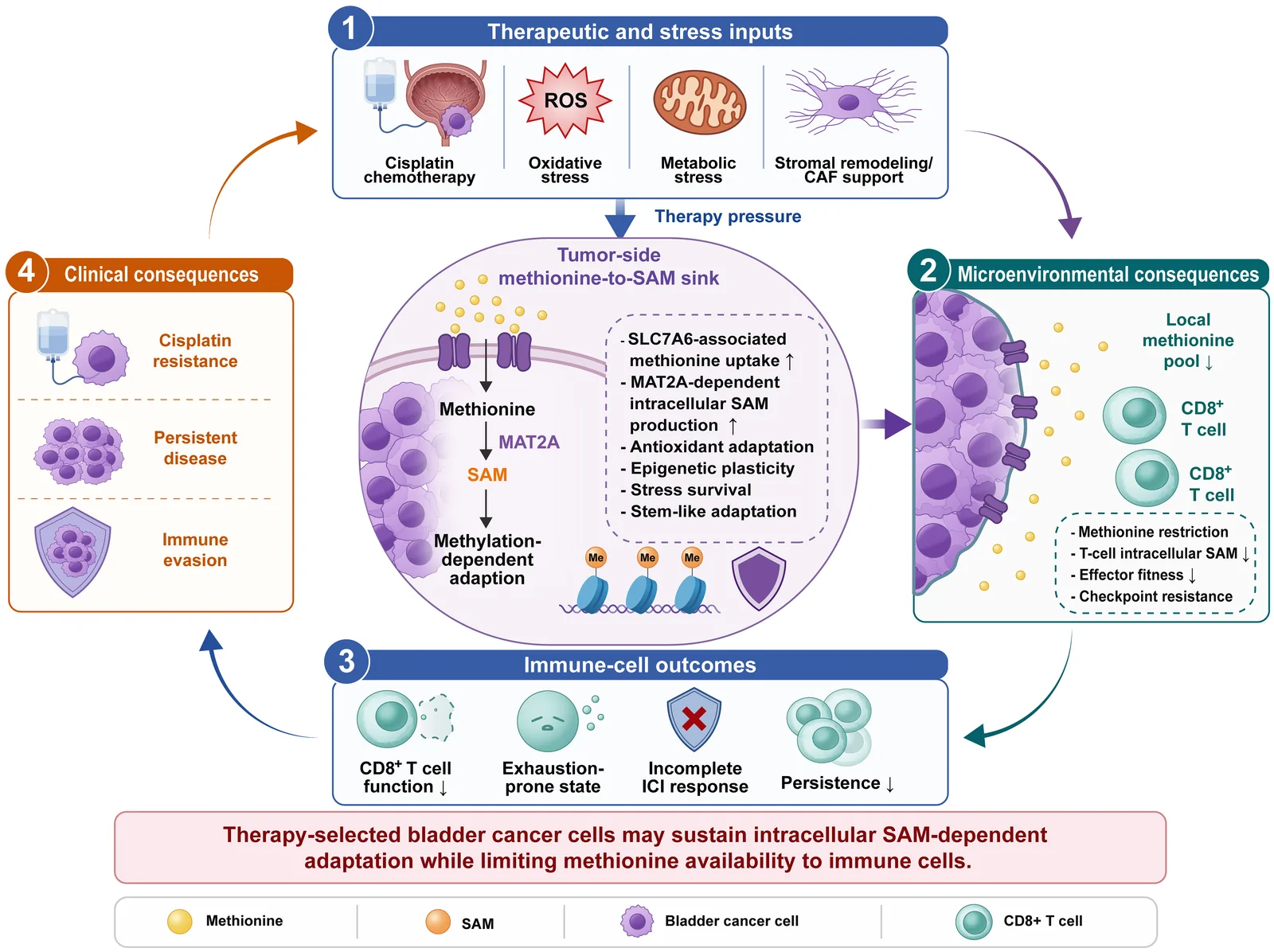
Proposed working model of platinum-selected methionine remodeling in bladder cancer. Cisplatin-resistant tumor cells may display two functionally distinct but convergent programs: MAT2A-dependent intracellular SAM production, which supports methylation-linked tumor adaptation, and SLC7A6-associated methionine uptake, which may reduce local methionine availability and constrain CD8+ T-cell fitness. The diagram is conceptual and does not imply that MAT2A directly mediates extracellular methionine depletion or that all depicted stress inputs and feedback relationships have been demonstrated in platinum-treated human tumors. ICI-related outcomes represent preclinical or translational inference. YTHDF1-associated immune escape is also influenced by methionine. However, this mechanism has not been shown to act downstream of the MAT2A and SLC7A6 programs associated with cisplatin resistance. We therefore discuss it as a parallel mechanism and do not depict it in this figure. CAF, cancer-associated fibroblast; CD8, cluster of differentiation 8; ICI, immune checkpoint inhibitor; MAT2A, methionine adenosyltransferase 2A; Me, methyl group; ROS, reactive oxygen species; SAM, S-adenosylmethionine; SLC7A6, solute carrier family 7 member 6; YTHDF1, YTH domain-containing family protein 1. ↑ and ↓ indicate increases and decreases, respectively.

## 4 How methionine competition constrains T-cell fitness and reinforces exhaustion

### 4.1 SAM deprivation erodes epigenetic fitness

Cross-tumor studies provide the mechanistic foundation for understanding the immune side of this checkpoint. SLC43A2-high cancer cells can deplete extracellular methionine and reduce methionine and SAM in CD8+ T cells, lowering H3K79me2, weakening STAT5 signaling, and impairing antitumor function (29). Accordingly, CD8+ T-cell presence does not necessarily indicate effective immunity, because a lymphocyte may reach the tumor without sufficient methyl-donor capacity to maintain its transcriptional and functional program. In hepatocellular carcinoma, elevated tumor SAM and 5′-methylthioadenosine (MTA) were associated with T-cell exhaustion. Exposure to either metabolite induced T-cell dysfunction *in vitro*, highlighting a mechanism distinct from intracellular methyl-donor deprivation (30).

### 4.2 Nutrient stress joins the exhaustion network

Methionine limitation does not act alone. In CD4+ T cells, cancer-cell methionine consumption reduces H3K79me2 and AMPK expression, increases endoplasmic-reticulum stress, and promotes progressive PD-1 expression (31). Early methionine availability during T-cell receptor signaling can also influence nonhistone methylation, NFAT1 activity, and later exhaustion propensity (32). Timing therefore matters as much as concentration. A transient shortage during initial activation may shape the later response differently from chronic deprivation in an established tumor.

T-cell exhaustion is maintained by persistent antigen, checkpoint signaling, transcriptional remodeling (33, 34), mitochondrial stress, and nutrient limitation (35–38). Methionine competition enters this network as a reinforcing pressure (39). It lowers the metabolic and epigenetic reserve of the T cell, making antigenic and checkpoint-mediated suppression more difficult to overcome. From this perspective, the methionine checkpoint operates both before and alongside PD-1, reducing the lymphocyte’s capacity to respond while strengthening tumor programs that resist recognition.

These observations should not be taken to mean that PD-1 upregulation alone defines a uniform exhausted state. Exhausted CD8+ T cells include TCF1+ progenitor, intermediate, and terminal compartments with distinct proliferative and cytotoxic properties (40). Bladder cancer studies have not yet established whether methionine limitation depletes progenitor cells, accelerates terminal differentiation, or produces reversible dysfunction across several states. One possibility is that methionine scarcity acts as a developmental gate, contracting the TCF1+ pool during priming or accelerating its transition toward terminal exhaustion. If correct, the same intervention could either weaken or preserve checkpoint responsiveness depending on when it is applied.

## 5 Discussion: therapeutic implications and compartment-specific risks

### 5.1 Weakening the tumor side of the checkpoint

MAT2A is the most pharmacologically advanced tumor-side target. The oral inhibitor AG-270/S095033 has reached phase I testing in advanced malignancies, primarily in tumors with homozygous MTAP deletion (41). The trial showed that MAT2A and SAM-related pharmacodynamics can be measured in patients, but it also reported reversible liver-test abnormalities, thrombocytopenia, anemia, and fatigue (41). These safety signals underscore the need to balance systemic target engagement against tolerability. Preclinical studies define how MAT2A inhibition disrupts PRMT5-dependent splicing and DNA-damage control in MTAP-deleted cells (42–44). In cisplatin-resistant bladder cancer, the therapeutic rationale is different: MAT2A inhibition is intended to collapse the intracellular SAM-dependent state that preserves stemness and treatment survival.

Transporter targeting addresses another layer, although the evidence differs by transporter and disease context. SLC7A6 is directly implicated in cisplatin-resistant bladder cancer, whereas SLC43A2 is supported across other tumors and participates in drug-resistance programs linked to methylation signaling (11, 29, 45). Blocking tumor methionine uptake could weaken the resistant cell and release nutrient pressure on infiltrating lymphocytes at the same time. Selectivity is crucial, however, because activated T cells require SLC7A5 for methionine uptake and broad amino-acid transport inhibition could reproduce the immune defect it is intended to correct (19).

### 5.2 The direction of intervention depends on the cell and treatment state

The wider one-carbon literature reveals the central therapeutic problem. Tumor MTHFD2 can promote PD-L1 expression, suggesting that suppression of tumor one-carbon flux may improve immune recognition (46). In contrast, formate supplementation can support CD8+ T-cell fitness and improve PD-1 blockade in preclinical models (47). The pathway can therefore point in opposite therapeutic directions: inhibiting methyl-donor metabolism may harm the tumor, while restoring the same metabolism may rescue the immune cell. The correct intervention cannot be inferred from the pathway name alone; it depends on which cell is targeted and at what stage of treatment.

Dietary and local depletion strategies expose the paradox most clearly. In cisplatin-resistant models, methionine restriction suppresses tumor adaptation, yet its benefit is reduced in an immunocompetent host, consistent with the dependence of antitumor lymphocytes on the same nutrient (11). In the YTHDF1 study, however, dietary restriction combined with PD-L1 blockade improves tumor control, whereas local bacterial methionine depletion enhances antitumor immunity (12). Studies in other tumor types provide further support for this approach. In preclinical models, dietary methionine restriction can enhance treatment responses (48) and alter m^6^A-mediated immune checkpoint regulation (49). Intermittent restriction can also promote ferroptosis in tumor cells and act synergistically with immune checkpoint blockade (50). However, methionine restriction affects tumor and immune cells differently, so its effects depend on the cellular context and tumor stage (51). A small clinical study has also assessed the feasibility of combining methionine restriction with chemotherapy in metastatic colorectal cancer (52).

The opposite intervention can also be beneficial, because SAM or one-carbon support may restore lymphocyte fitness and improve immunotherapy in selected preclinical settings (47, 53). Systemic methyl-donor supplementation, however, may simultaneously support malignant-cell methylation, stress adaptation, and growth. The danger is not simply toxicity but helping the wrong cell. The most rational strategies are likely to be local, tumor-selective, or temporally controlled. Depending on treatment phase, tumor methionine acquisition could be restricted, methionine preserved during T-cell priming, or immune-cell methyl-donor fitness restored after tumor uptake has been blocked.

### 5.3 Patient selection and combination therapy

Methionine-directed therapy should not be applied to bladder cancer as a uniform metabolic phenotype. A treatment-state signature based on low circARHGAP10, high MAT2A, and high SLC7A6 may identify tumors selected by platinum exposure. Immune pharmacodynamic assessment should integrate pathway readouts with T-cell effector function, metabolic fitness, and differentiation state. Candidate progenitor or memory-like programs should be distinguished from terminal dysfunction (40), and exhaustion-associated markers should be interpreted across cellular states rather than used as standalone proof of exhaustion (54). YTHDF1, RIG-I, and PD-L1 may define a complementary immune-visibility module. MTAP/CDKN2A loss identifies a separate genotype-selected dependency linked to MTA accumulation and PRMT5 biology (55–57), but this dependency should not be treated as equivalent to the state selected by cisplatin.

The most informative experiments will combine these compartments rather than study them separately. Paired pre- and post-platinum tumors should be analyzed by spatial metabolomics and multiplex imaging to determine whether MAT2A- or SLC7A6-high regions overlap with methionine-low, H3K79me2-low, functionally impaired T-cell niches. Stable-isotope tracing and sorted-cell metabolomics can define the direction of methionine flux. Patient-derived organoids combined with autologous immune cells can then compare MAT2A inhibition, selective transporter perturbation, transient methionine restriction, and ICI combinations. The therapeutic goal is not merely tumor shrinkage, but weakening the resistant tumor while preserving the immune cell needed to complete the response.

### 5.4 Limitations, alternative explanations, and translational risks

The proposed checkpoint is a unifying biological model rather than a claim that every component forms one obligatory molecular chain. Methionine also influences immune escape through YTHDF1–RIG-I/PD-L1 signaling. However, this mechanism has not been shown to act downstream of the cisplatin-selected MAT2A and SLC7A6 programs. We therefore discuss it as a parallel methionine-sensitive mechanism. Conditioned-medium effects may include metabolites and soluble factors beyond methionine; transporter activity may vary across treatment states; and YTHDF1 regulates transcripts beyond RIG-I and PD-L1. An additional mechanism operates through YTHDF2-mediated DDX58/RIG-I mRNA decay, which suppresses innate immunity, limits CD8+ T-cell recruitment, and reduces BCG responsiveness in bladder-cancer models (13). RIG-I stability is also controlled by asparagine-driven, CBL-mediated proteasomal degradation in bladder cancer, indicating that this innate-sensing node is metabolically convergent rather than uniquely methionine-defined (58). Nevertheless, the convergence of therapy-selected MAT2A dependence, SLC7A6-associated nutrient competition, and methionine-sensitive immune-evasion programs provides a testable explanation for how metabolic adaptation can connect cisplatin resistance with immune dysfunction. Paired pre- and post-platinum specimens should be used to define treatment-state dependence, spatial organization, and biomarkers of response to compartment-selective intervention.

Shared metabolic dependence creates several translational risks, because systemic MAT2A inhibition may alter methyl-donor availability beyond the tumor and broad transporter blockade may disrupt T-cell methionine uptake (19, 41). Dietary restriction therefore requires monitoring of immune fitness and nutritional reserve, particularly in frail patients (11, 48). Conversely, SAM restoration could support malignant-cell methylation and plasticity even when immune fitness improves (14–16). Compensatory flux through serine-glycine and folate metabolism, transsulfuration, methionine salvage, or alternative transporters could also limit durability (14–16). These risks favor tumor-selective delivery, treatment-state biomarkers, and immune and nutritional monitoring. Intermittent scheduling may outperform continuous restriction by separating tumor-directed methionine depletion from T-cell priming or reinvigoration. The principal evidence layers, translational entry points, and unresolved questions are summarized in **Table 1**.

**Table 1:** Mechanistic layers of the therapy-induced methionine checkpoint.

Evidence layer	Mechanism or context	Biological consequence	Therapeutic implication and unresolved question
Therapy-selected tumor adaptation (11, 18)	CircARHGAP10 levels ↓; TRIM25-mediated MAT2A degradation ↓; MAT2A stability ↑	Reduced circARHGAP10 levels decrease TRIM25-mediated MAT2A ubiquitination and degradation, stabilizing MAT2A and sustaining SAM-related methylation, stem-like renewal, and cisplatin resistance	MAT2A inhibition may collapse the resistant state; paired pre- and post-platinum validation is required
Tumor-immune nutrient competition (11, 29)	SLC7A6-associated methionine uptake ↑	SLC7A6-associated uptake lowers CD8+ T-cell methionine/SAM, H3K79me2, pSTAT5, and effector function	Tumor-selective transporter inhibition may relieve competition; selective pharmacology and human spatial flux remain needed
Reduced innate immune sensing (12)	Methionine availability ↑; YTHDF1 expression ↑; RIG-I/MAVS signaling ↓	YTHDF1 promotes the loss of RIG-I transcripts, reducing type I interferon signaling	Local methionine depletion or YTHDF1 targeting may restore innate sensing; clinical validation is needed
Enhanced PD-L1-mediated immune evasion (12, 49)	Methionine availability ↑; YTHDF1 expression ↑; eIF5B-dependent PD-L1 translation ↑	YTHDF1 interacts with eIF5B to enhance PD-L1 translation and promote immune evasion	Local methionine depletion or YTHDF1 targeting may improve ICI response; delivery, safety, and clinical relevance require validation
Mechanistic reinforcement across tumors (29, 32, 39, 46)	Transport competition, methylation, exhaustion, and other one-carbon nodes	Transport competition and methyl-donor loss connect nutrient stress with epigenetic and functional T-cell exhaustion	Timing and cellular compartment determine whether metabolic restriction suppresses tumor cells or harms lymphocytes
Translational entry points (41, 49, 57)	MAT2A inhibition, dietary intervention, and MTAP-directed strategies	MAT2A inhibition, dietary or local methionine modulation, and MTAP-directed strategies provide complementary routes	The central challenge is to weaken tumor adaptation without compromising antitumor immunity

Citations are representative rather than exhaustive.

CD8, cluster of differentiation 8; circARHGAP10, circular RNA derived from the Rho GTPase activating protein 10 (ARHGAP10) gene; eIF5B, eukaryotic translation initiation factor 5B; H3K79me2, histone H3 lysine 79 dimethylation; ICI, immune checkpoint inhibitor; MAT2A, methionine adenosyltransferase 2A; MAVS, mitochondrial antiviral-signaling protein; MTAP, methylthioadenosine phosphorylase; PD-L1, programmed death-ligand 1; pSTAT5, phosphorylated signal transducer and activator of transcription 5; RIG-I, retinoic acid-inducible gene I; SAM, S-adenosylmethionine; SLC7A6, solute carrier family 7 member 6; TRIM25, tripartite motif-containing protein 25; YTHDF1, YTH domain-containing family protein 1. ↑ and ↓ indicate increases and decreases, respectively, in the specified parameter within the relevant study context.

## 6 Conclusion

Therapy-induced methionine remodeling offers a testable framework for linking platinum resistance to immune dysfunction in bladder cancer. In cisplatin-resistant models, MAT2A-dependent SAM maintenance supports tumor-cell fitness, whereas SLC7A6-associated methionine competition lowers CD8+ T-cell methionine/SAM and weakens H3K79me2-STAT5 signaling. A separate evidence stream connects methionine-sensitive YTHDF1 signaling to reduced RIG-I-mediated immune sensing and increased PD-L1 translation. Testing this framework will require paired pre- and post-platinum sampling, spatial and cell-resolved analysis, and compartment-selective intervention. Methionine-directed therapy should therefore be guided by treatment state, cellular target, and therapeutic timing.
